# Hereditary angioedema due to C1 inhibitor deficiency: real-world experience from the Icatibant Outcome Survey in Spain

**DOI:** 10.1186/s13223-021-00641-3

**Published:** 2021-12-29

**Authors:** Mar Guilarte, Anna Sala-Cunill, María Luisa Baeza, Rosario Cabañas, María Dolores Hernández, Ethel Ibañez, Carlos Hernando de Larramendi, Ramon Lleonart, Teófilo Lobera, Luis Marqués, Blanca Sáenz de San Pedro, Jaco Botha, Irmgard Andresen, Teresa Caballero, for the IOS Study Group

**Affiliations:** 1 Allergy Section, Medicine Department, Hospital Universitari Vall d’Hebron, Barcelona, Spain; 2 Spanish Research Network on Allergy (National Allergy Network ARADyAL: Asma, Reacciones Adversas y Alérgicas), Instituto de Salud Carlos III, Madrid, Spain; 3 Vall d’Hebron Institut de Recerca, Hospital Universitari Vall d’Hebron, Barcelona, Spain; 4 Allergy Department, Hospital General Universitario Gregorio Marañón, Madrid, Spain; 5 Biomedical Research Network on Rare Diseases, CIBERER-U761, Gregorio Marañón Health Research Institute, Madrid, Spain; 6 Allergy Department, Hospital Universitario La Paz, Hospital La Paz Institute for Health Research (IdiPaz), Madrid, Spain; 7 Biomedical Research Network on Rare Diseases, CIBERER-U754, Madrid, Spain; 8 Allergy Department, IIS-Hospital Universitari I Politècnic La Fe, Valencia, Spain; 9 Allergy Section, Hospital Marina Baixa, Villajoyosa and Centro de Especialidades Foietes, Benidorm, Spain; 10 Allergy Unit, Medicine Department, IDIBELL, Hospital Universitari de Bellvitge, L’Hospitalet de Llobregat, Barcelona, Spain; 11 Allergy Department, Hospital San Pedro, Logroño, Spain; 12 Allergy Section, IRBLleida, Hospital Universitari Santa Maria and Hospital Universitari Arnau de Vilanova, Lleida, Spain; 13 Allergy Section, Hospital Universitario de Jaén, Jaén, Spain; 14 Takeda Pharmaceuticals International AG, Zurich, Switzerland

**Keywords:** Hereditary angioedema, Registries, Spain, Bradykinin, Bradykinin B_2_ receptor antagonists, Icatibant, On-demand treatment

## Abstract

**Background:**

The Icatibant Outcome Survey (IOS) is an international registry monitoring the use of icatibant, a bradykinin B_2_ receptor antagonist indicated for the acute treatment of hereditary angioedema (HAE) attacks. Our goal was to assess disease characteristics and icatibant treatment outcomes in patients with HAE due to C1 inhibitor deficiency (HAE type 1 or 2 (HAE-1/2)) from Spain relative to other countries participating in IOS.

**Methods:**

Descriptive retrospective analyses of data are reported from 10 centers in Spain vs 51 centers in 12 other participating countries (July 2009 to January 2019).

**Results:**

No meaningful differences were identified between patients in Spain (n = 119) and patients across other countries (n = 907) regarding median age at symptom onset (15.0 vs 12.0 years) or diagnosis (22.3 vs 20.5 years). Overall HAE attack rates (total attacks/total years of follow-up) were 2.66 in Spain and 1.46 across other countries. Patients in Spain reported fewer severe/very severe HAE attacks before treatment (41.0% vs 45.9%; *P* < 0.0001) and, for icatibant-treated attacks, longer median time to treatment (2.9 vs 1.0 h), time to attack resolution (18.0 vs 5.5 h), and total attack duration (24.6 vs 8.0 h). Use of androgens for long-term prophylaxis was higher in Spain (51.2% vs 26.7%).

**Conclusion:**

Patients with HAE-1/2 in Spain reported fewer severe/very severe attacks, administered icatibant later, and had longer-lasting attacks than did patients across other countries in IOS. These differences may indicate varying disease management practices (e.g., delayed icatibant treatment) and reporting. Efforts to raise awareness on the benefits of early on-demand treatment may be warranted.

*Trial registration*: NCT01034969.

**Supplementary Information:**

The online version contains supplementary material available at 10.1186/s13223-021-00641-3.

## Background

Hereditary angioedema due to C1 inhibitor deficiency or dysfunction (HAE type 1 or type 2 (HAE-1/2)) is a rare and potentially life-threatening disease affecting ~ 1 in 50,000 individuals worldwide [1]; the reported minimum prevalence in Spain is ≥ 1.09 per 100,000 people [2]. HAE-1/2 is characterized by swelling of the skin and mucous membranes, causing considerable pain and temporary disability, with attacks recurring with unpredictable frequency, severity, and location [1, 3]. Edematous attacks in the upper airways can be fatal due to risk of asphyxiation [4, 5]. Due to its rarity and overlapping symptoms with other diseases, misdiagnoses and delays in diagnosis have been frequently reported [3, 6, 7].

International HAE management guidelines recommend that on-demand treatment be considered for all HAE attacks; patients be provided with sufficient on-demand medication to treat two attacks; and patients who are prescribed treatment licensed for self-administration be taught to self-administer, facilitating early treatment and optimal response [1]. Icatibant is a subcutaneously administered bradykinin B_2_ receptor antagonist for symptomatic treatment of acute attacks in adult patients with HAE [8]. In Europe, icatibant is also approved for use in pediatric patients aged ≥ 2 years [9].

The Icatibant Outcome Survey (IOS; NCT01034969), initiated in 2009, is an ongoing, international, prospective, observational registry that monitors real-world use of icatibant for acute treatment of angioedema attacks [10, 11]. The objective of the current analysis was to compare disease characteristics and icatibant treatment outcomes in patients with HAE-1/2 from centers in Spain with those from centers in the 12 other countries participating in IOS.

## Patients and methods

### Study design and patients

This is a descriptive, retrospective analysis of IOS patient data from 10 centers in Spain vs 51 centers in Australia, Austria, Brazil, Czech Republic, Denmark, France, Germany, Greece, Israel, Italy, Sweden, and the United Kingdom; data were collected from July 2009 to January 2019. The design of IOS has been previously described [10, 12, 13]. IOS is conducted in accordance with the Declaration of Helsinki and the International Conference on Harmonisation Good Clinical Practice Guidelines, and approval was granted by health authorities and local ethics committees. Written informed consent was obtained from all enrolled patients.

Only patients diagnosed with HAE-1/2 were included in the current analysis. Data were collected from patients at time of IOS enrollment (based on prestudy experience) and then at follow-up visits every ~ 6 months. Patients reported characteristics of treated attacks (with any kind of treatment) during follow-up, including location, severity, and triggers/prodromes. Attack severity was evaluated according to interference with daily activities. Icatibant treatment outcomes were retrieved from patients with complete attack outcome data for time to icatibant treatment (i.e., time from attack onset to first icatibant injection), time to complete symptom resolution (i.e., time from first icatibant dose to complete symptom resolution), and total attack duration (i.e., time from attack onset to complete symptom resolution) for HAE attacks that were treated with icatibant.

### Statistical analyses

Data are presented as median (interquartile range (IQR)) or proportion of patients in each group (Spain vs all other participating countries), unless otherwise specified. Importantly, as these are post hoc analyses, a formal significance level was not defined, and no adjustments were made for multiple comparisons. *P*-values should be viewed as descriptive statistics providing an indication of the difference between quantities/distributions. Pearson’s chi-squared test was used to compare categorical distributions. Attack severity (very mild/mild/moderate vs severe/very severe) was compared using a generalized linear model for repeated measures.

## Results

### Patient demographics and baseline characteristics in Spain vs other countries participating in IOS

A total of 1026 patients with HAE-1/2 provided demographic data: 119 patients from Spain and 907 patients from the 12 other IOS countries (Table 1). A larger proportion of Spanish patients were enrolled by 2011 compared with other countries combined (Additional file 1: Fig. S1A), resulting in longer median duration of follow-up at the time of data cutoff (5.6 years in Spain vs 2.4 years in other countries). Follow-up duration of ≥ 5 years was recorded for 55.5% of patients in Spain and 24.1% in other countries, and ≥ 1 year was recorded for 79.0% and 65. 2%, respectively (Additional file 1: Fig. S1B).

**Table 1:** Patient demographics and baseline characteristics

Characteristic	Spain n = 119	Other Countries^a^ n = 907
Sex, n (%)		
Female	64 (53.8)	548 (60.4)
Male	55 (46.2)	359 (39.6)
HAE type, n (%)		
HAE-1	114 (95.8)	838 (92.4)
HAE-2	5 (4.2)	69 (7.6)
Median (IQR) age at first symptoms, y	15.0 (5.0–20.0)	12.0 (5.0–18.0)
Median (IQR) age at diagnosis, y	22.3 (13.6–36.0)	20.5 (12.6–32.9)
Median (IQR) time to diagnosis, y^b^	6.4 (1.0–18.3)	5.4 (0.2–17.0)
Median (IQR) age at enrollment, y	39.3 (27.2–50.4)	39.0 (27.5–51.8)
Median (IQR) age at data extract, y	45.0 (34.0–56.0)	44.0 (32.0–57.0)
Median (IQR) duration of follow-up in IOS, y	5.6 (1.2–7.0)	2.4 (0.4–4.9)

*HAE-1/2* HAE due to C1 inhibitor deficiency, *IOS* Icatibant Outcome Survey

^a^Australia, Austria, Brazil, Czech Republic, Denmark, France, Germany, Greece, Israel, Italy, Sweden, and the United Kingdom

^b^Defined as the difference between age at first symptoms and age at diagnosis

**Fig. 1:**
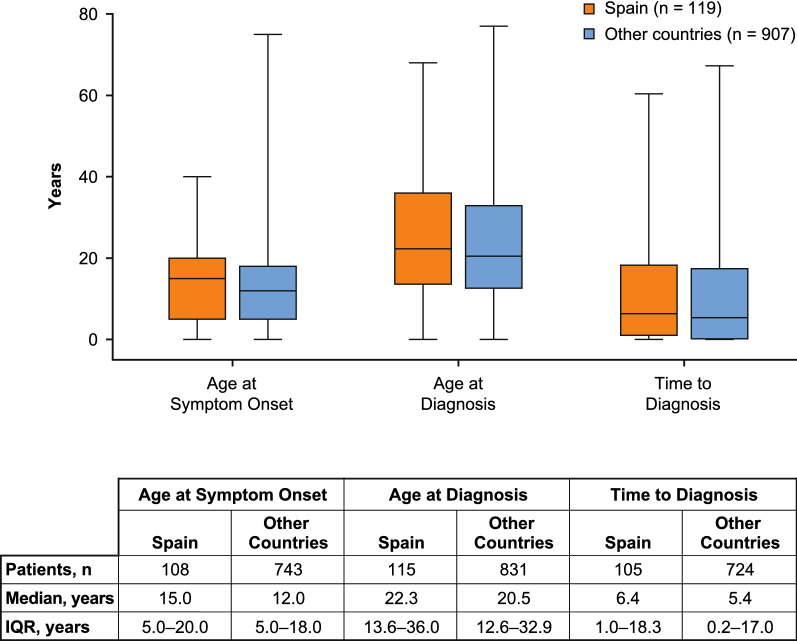
Age at symptom onset, age at diagnosis, and time to diagnosis in patients with HAE-1/2 from Spain and from other countries participating in IOS. Lines within box plots represent median age in years, lower and upper lines of boxes represent the 25th and 75th percentiles, and box whiskers denote the minimum and maximum. The minimum time from symptom onset to diagnosis was −19.0 years in patients from Spain and −41.8 years in patients from other countries for patients diagnosed before symptom onset based on family history, genetic mutation analysis, concentration and/or functional testing of C1 inhibitor, or concentration of C4. *HAE-1/2* hereditary angioedema due to C1 inhibitor deficiency, *IOS* Icatibant Outcome Survey, *IQR* interquartile range

No meaningful differences were identified in the proportion of females (53.8% vs 60.4%) or median age at IOS enrollment (39.3 (IQR 27.2–50.4) vs 39.0 (IQR 27.5–51.8) years) between Spanish patients and patients from other IOS countries. Consistent with the general HAE population, > 92% of patients in both groups were diagnosed with HAE-1 (Table 1). There were no meaningful differences regarding median age at symptom onset, median age at diagnosis, or median time to diagnosis (Fig. 1). By year of birth, median time to diagnosis suggested that substantial improvements in HAE diagnosis had been made for patients born from 1970 onward in Spain, and from 1975 onward across other countries (Additional file 1: Fig. S2). The greatest improvement in time to diagnosis in Spain was observed for patients born during 1970–1974 (median 3.0 years) compared with those born during 1965–1969 (median 12.5 years). In contrast, improvements in time to diagnosis across other countries were more gradual.

### Socioeconomic burden of HAE-1/2 in Spain compared with other countries: employment status, missed work/education, and hospitalizations

Employment status at IOS entry was available for 97 patients from Spain and 641 patients from other countries (Additional file 1: Table S1). Proportions of patients were similar with respect to employed (62.9% vs 55.7%) and unemployed status (9.3% vs 7.2%).

In the 12 months before IOS entry, a higher percentage of patients from Spain missed no days of work or education (76.9% (40/52)) than from other countries (65.3% (231/354)). Of patients who had missed ≥ 1 day, proportions were similar for 1–7 missed days (21.2% vs 19.2%) but lower in Spain for > 7 missed days (1.9% vs 15.5%).

Likewise, during follow-up in IOS, a higher percentage of patients from Spain reported no missed days 83.8% (62/74) vs 72.9% (285/391) patients from other countries. Trends in the proportions of patients with 1–7 missed days (12.2% vs 15.1%) and > 7 missed days (4.1% vs 12.0%) were similar to before IOS entry.

In the 12 months before IOS entry, fewer patients from Spain were hospitalized compared with patients from other countries; 96.8% and 85.5%, respectively, reported no hospitalizations. Between one and five hospitalizations were reported by 3.2% and 13.6%, respectively; more than five hospitalizations were reported by 0.0% and 1.0%, respectively.

### Characteristics of treated HAE attacks during follow-up in IOS

#### Total number of treated attacks (with any treatment) and untreated attacks

An overall incidence rate of HAE attacks was calculated from total number of reported attacks and total number of years of follow-up; for all patients, incidence rates during the follow-up period were 2.66 in Spain and 1.46 in other countries. During follow-up, a total of 1662 HAE attacks in 101 patients from Spain and 5645 attacks in 464 patients from other countries were recorded as treated (with any type of treatment); median (IQR) number of treated attacks per patient was 10.0 (3.0–19.0) in Spain and 5.0 (2.0–12.5) in other countries. There were 77/107 (72.0%) and 293/548 (53.5%) patients in Spain and other countries, respectively, who reported ≥ 1 untreated attack (without any kind of treatment); the median (IQR) number of untreated attacks per patient during follow-up was 5.0 (0.0–17.0) in Spain compared with 1.0 (0.0–7.0) in other countries.

#### Location of treated attacks

Attack location data were available for 1595 treated attacks in patients from Spain and 5407 treated attacks in patients from other countries. The vast majority of these attacks affected a single site in both groups, with 189 (11.8%) and 590 (10.9%) attacks involving multiple sites, respectively. Overall, proportions of patients were generally comparable with respect to attacks affecting the abdomen (54.4% vs 58.0%), skin (47.8% vs 43.5%), larynx (3.9% vs 5.0%), and other locations (6.0% vs 4.8%).

#### Severity of attacks before treatment

**Fig. 2:**
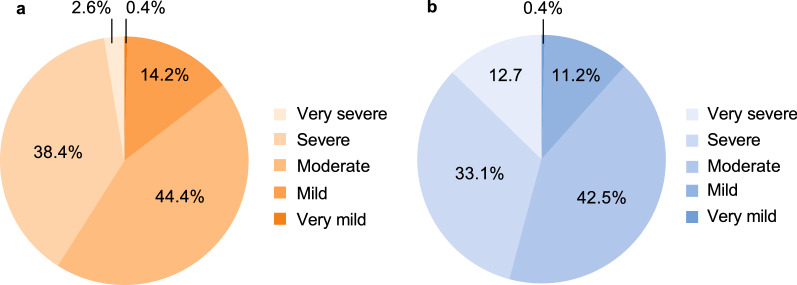
Severity of HAE attacks before treatment for patients in IOS with available data on this parameter in **a** Spain (1215 attacks) and **b** other countries (4787 attacks). *P* < 0.0001 severe/very severe attacks for Spain vs other IOS countries. *HAE* hereditary angioedema, *IOS* Icatibant Outcome Survey

Patients from Spain were significantly less likely to report severe/very severe attacks compared with patients from other countries (498/1215 (41.0%) vs 2195/4787 (45.9%) attacks; *P* < 0.0001; Fig. 2), mainly due to a lower number of very severe attacks.

#### Triggers and prodromal symptoms for treated attacks

The most frequent triggers (reported by ≥ 2% of patients in either group) were similar between patients from Spain (1662 attacks) and patients from other countries (5645 attacks), and included emotional distress (11.0% vs 9.2%), estrogen level changes in females (6.7% (based on 1040 attacks) vs 4.2% (based on 3602 attacks)), physical trauma (3.8% vs 2.5%), and infection (3.7% vs 2.7%).

The most frequent prodromal symptoms (reported by ≥ 2% of patients in either group; out of 1662 and 5645 attacks for patients from Spain and those from other countries, respectively) were tiredness (2.0% vs 5.8%), erythema marginatum (2.7% vs 3.5%), tightness/prickling sensation in the skin (1.3% vs 2.7%), and nausea (1.1% vs 2.8%).

### Icatibant-treated attacks and treatment outcomes

#### Number of patients and icatibant-treated attacks

Of patients with any treated attacks, 93/101 (92.1%) patients from Spain and 428/464 (92.2%) patients from other countries received icatibant for at least one attack. Icatibant was used for 1203/1568 (76.7%) treated attacks in patients from Spain, and in 4472/5233 (85.5%) treated attacks in patients from other countries. Median (IQR) number of icatibant-treated attacks per patient was 8.0 (3.0–15.0) and 4.0 (2.0–11.0), respectively. The remaining 365 (23.3%) and 761 (14.5%) attacks in each group, respectively, received treatments other than icatibant. Proportions of patients over time with attacks receiving at least one icatibant injection and attacks with only other treatments were similar across the two groups (Additional file 1: Fig. S3).

#### Time to icatibant treatment, time to symptom resolution, and total attack duration during follow-up

**Fig. 3:**
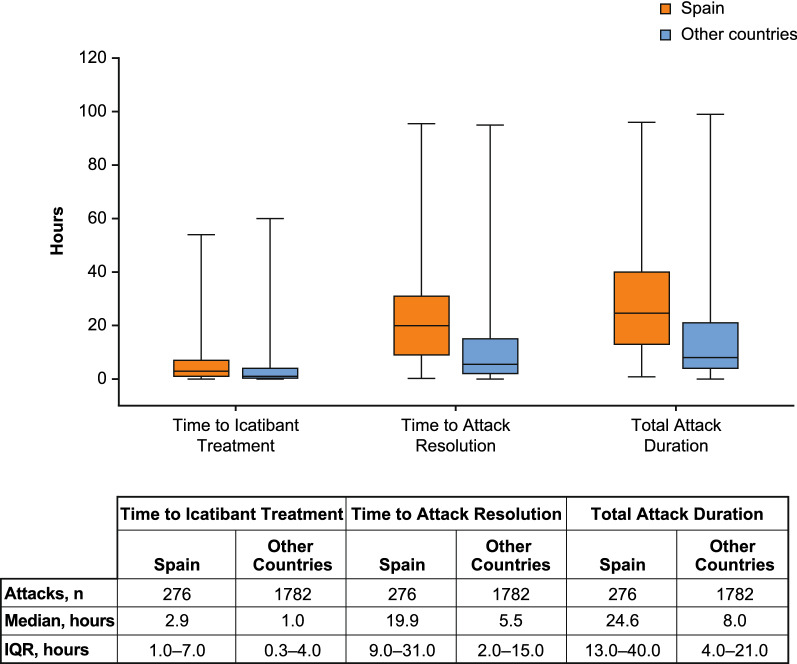
Time to icatibant treatment, time to attack resolution, and attack duration (in patients having available data for all three parameters). Lines within box plots represent median time in h, lower and upper lines of boxes represent the 25th and 75th percentiles, and box whiskers denote the minimum and maximum. *IQR* interquartile range

Time to icatibant treatment was longer in patients from Spain compared with other countries (median 2.9 vs 1.0 h; Fig. 3). A higher proportion of attacks in patients from Spain had a time to icatibant treatment of ≥ 2 h (54.5% vs 34.3%) and > 5 h (34.4% vs 19.4%).

Time to attack resolution was longer in patients from Spain compared with patients from other countries (median 18.0 vs 5.5 h), with a greater proportion of attacks with time to resolution of > 5 h (79.8% vs 51.5%). Total attack duration was also longer in patients from Spain (median 24.6 vs 8.0 h), with a higher proportion of attacks lasting > 4 h (93.8% vs 73.0%).

### Self-administration of icatibant

Data on type of administration (self-administered or health-professional administered) were available for 1024 attacks in 89 patients from Spain and 3953 attacks in 378 patients from other countries (Table 2). Overall, self-administered icatibant (by patients themselves or a caregiver) was used to treat 93.7% and 95.7% of attacks in each group, respectively. The proportions of patients using self-administration for their first attack during IOS follow-up were comparable in the two groups (82.0% vs 85.7%). For patients in Spain, the proportion treated with self-administered icatibant increased from 82.0% for the first attack to 92.2% for the third attack.

**Table 2:** Number of HAE attacks treated with icatibant by self-administration compared with icatibant administered by a health care professional

	Spain n = 119	Other countries n = 907
HAE attacks treated with icatibant with administration data	1024	3953
Attacks for which patients self-administered icatibant, n (%)^a^	959 (93.7)	3783 (95.7)
Attacks treated with icatibant administered by a health care professional, n (%)^a^	65 (6.3)	174 (4.4)

Data on type of administration per attack were available for 89 patients in Spain and 378 patients in other countries

*HAE* hereditary angioedema

^a^Administrations by a family member or other non-health care professional caregiver are included in the self-administration category, and all non-self-administrations are included in health care professional category; data shown here correspond to those attacks with available data on who administered the treatment

More detailed analyses showed that self-administration in Spain lagged behind other countries during the first 3 years of IOS, but rates were then comparable from 2012 onward (Additional file 1: Fig. S4).

### Icatibant treatment outcomes over time and by patient/attack characteristic

Median time to symptom resolution was shorter during 2009–2012 and longer during 2013–2019 in patients from Spain than from other countries. Consequently, median total attack duration was longer during 2013–2019 in patients from Spain as well (Additional file 1: Fig. S5).

Median time to treatment was longer in patients from Spain from 2009–2019. When time to treatment was stratified as < 5 vs ≥ 5 h from attack onset, greater proportions of patients in Spain treated attacks later in most of the subgroup categories (i.e., patient sex and age, attack severity and location; Additional file 1: Fig. S6).

### Use of long-term prophylaxis and effect on icatibant-treated attacks

Use of long-term prophylaxis (LTP) was reported for 80 (67.2%) patients in Spain and 470 (51.8%) patients across other countries. Commonly used LTP medications were similar in patients from Spain compared with other countries, except for a proportionally greater use of anabolic steroids in Spain (Table 3); more recently approved LTP options—lanadelumab and subcutaneous C1 inhibitor—were not available in the participating countries during follow-up. Per year, proportions of patients who reported using LTP remained consistent from 2010 onward, with greater use of LTP reported in Spain compared with other countries (Additional file 1: Fig. S7).

**Table 3:** Ongoing long-term prophylaxis medications for HAE reported in patients enrolled in IOS

Medication, n (%)	Spain n = 73	Other countries N = 385
Danazol	29 (39.7)	215 (55.8)
Stanozolol	32 (43.8)	19 (4.9)
Plasma-derived C1 inhibitor^a^	21 (28.8)	87 (22.6)
Tranexamic acid	19 (26.0)	102 (26.5)
Oxandrolone	0	8 (2.1)
Recombinant C1 inhibitor	0	5 (1.3)
Other	5 (6.8)	47 (12.2)

All long-term prophylaxis medications reported at any time during follow-up in IOS are included in the summary; patients may be included in more than one category

*HAE* hereditary angioedema, *IOS* Icatibant Outcome Survey

^a^Includes Berinert®, Cinryze®, and unspecified C1 inhibitors

For patients with recorded LTP use who subsequently ceased using LTP, overall rates of attacks (total attacks divided by total years) while LTP was in use were 0.74 in Spain and 2.47 across other countries. Following cessation of LTP, incidence rates were 1.42 and 3.09, respectively.

In Spain, severity of attacks in patients with and without LTP use was comparable; severe/very severe attacks were reported in 298/738 (40.4%) attacks with LTP compared with 199/470 (42.3%) attacks without LTP. Across other countries, severe/very severe attacks were reported in 1057/1938 (54.5%) attacks with LTP and 1124/2833 (39.7%) attacks without LTP; the proportion of moderate attacks with and without LTP was 45.1% and 43.0%, respectively, for patients in Spain, and 33.0% and 49.2%, respectively, across other countries.

### Safety of icatibant in treated patients

Overall, adverse events (AEs) were reported in 35.8% of patients from Spain and 28.9% of patients from other countries. AEs considered related to icatibant were reported in 2.1% and 4.4% of patients, respectively (Table 4). The most frequently reported icatibant-related AEs in patients from Spain were asthenia (seven events), hypersensitivity (six events), and administration site reaction (four events), all of which were reported in one patient each. The six hypersensitivity events all occurred in the same patient. The patient reported itching, burning, and erythema on the abdomen after icatibant administration.

**Table 4:** AEs in icatibant-treated patients that were considered related to icatibant by investigators, by Preferred Term

	Spain n = 95		Other countries n = 498	
	Patients, n (%)	Events, n	Patients, n (%)	Events, n
Any AE	34 (35.8)	95	144 (28.9)	302
Any icatibant-related AE	2 (2.1)	18	22 (4.4)	57
Administration site reaction	1 (1.1)	4	0	0
Infusion site pain	1 (1.1)	1	1 (0.2)	1
Asthenia	1 (1.1)	7	0	0
Hypersensitivity	1 (1.1)	6	0	0
Infusion site erythema	0	0	11 (2.2)	22
Hyperemia	0	0	3 (0.6)	4
Application site erythema	0	0	3 (0.6)	3
Pain	0	0	3 (0.6)	3
Application site pain	0	0	2 (0.4)	3
Drug ineffective	0	0	2 (0.4)	2
Blood pressure decreased	0	0	1 (0.2)	4
Gastritis	0	0	1 (0.2)	3
Abdominal pain upper	0	0	1 (0.2)	1
Hiatus hernia	0	0	1 (0.2)	1
Nausea	0	0	1 (0.2)	1
Injection site hemorrhage	0	0	1 (0.2)	1
Injection site reaction	0	0	1 (0.2)	1
Angioedema	0	0	1 (0.2)	1
Localized edema	0	0	1 (0.2)	1
Edema	0	0	1 (0.2)	1
Therapeutic product ineffective	0	0	1 (0.2)	1
Herpes zoster	0	0	1 (0.2)	1
Depression	0	0	1 (0.2)	1
Hot flush	0	0	1 (0.2)	1
Any serious AE	18 (18.9)	34	79 (15.9)	126
Any icatibant-related serious AE				
Gastritis	0	0	1 (0.2)	1
Angioedema	0	0	1 (0.2)	1^a^

*AE* adverse event

^a^Two doses of icatibant were administered before this patient was hospitalized for 24 h; in the hospital, fresh frozen plasma was given, and the patient was discharged the following day without any sequelae

Serious AEs (related and unrelated to icatibant) were reported in 18.9% of patients from Spain and 15.9% of patients from other countries. Two serious AEs, one event each of gastritis and angioedema, were considered related to icatibant by investigators; neither of these events was reported in patients from Spain.

## Discussion

This analysis of data from 1026 patients with HAE-1/2 enrolled in IOS provides valuable insights into patient characteristics, treatment patterns, and icatibant treatment outcomes in Spain compared with other IOS countries. General disease characteristics of HAE-1/2 were similar across both groups, with no meaningful differences with respect to age at symptom onset, time to diagnosis, attack location, and most frequently reported triggers and prodromal symptoms. Numbers of treated and untreated attacks per patient were higher in Spain compared with other countries, as was overall incidence rate of attacks, indicating that patients in Spain experienced more frequent HAE attacks during the follow-up period. Over 90% of patients in both groups who reported attacks and received treatment used icatibant during follow-up, with a median number of 8.0 icatibant-treated attacks per patient in Spain compared with 4.0 attacks per patient across other countries. The safety profile of icatibant was comparable in both groups and consistent with previous findings.

Patients in Spain reported significantly fewer severe/very severe attacks before treatment compared with other countries. This finding is consistent with a previous comparison of IOS data where patients in Spain, Germany, and Austria reported fewer severe/very severe attacks compared with patients in France, Italy, and the United Kingdom [13]. We also observed that in Spain, time to treatment with icatibant after onset of attack was longer than for patients in other countries, likely contributing to the longer time to attack resolution and total attack duration.

International guidelines strongly recommend treating HAE attacks as early as possible for optimal treatment response [1], and shorter time to icatibant treatment has been associated with shorter attack duration [10]. The current findings suggest that educating health care professionals, patients, and caregivers in Spain on the importance of early on-demand treatment of HAE attacks may improve outcomes. Self-administration of on-demand treatment is recommended to facilitate early treatment. Use of self-administered icatibant overall and its use over time was consistent with previous findings [13, 14], with > 90% of attacks self-treated from 2012 onward. In Spain, self-administration rate increased with number of attacks treated, suggesting increasing patient confidence with greater use.

Although patients in Spain reported a higher frequency of attacks, treated attacks later, and experienced longer-lasting attacks, they also reported fewer days of missed work/education, and socioeconomic burden was consistent with pre-IOS entry data. Combined with the observation of fewer severe/very severe attacks in patients in Spain, this finding suggests less severe disease and/or disease burden compared with patients in other countries. However, only a portion of IOS survey participants provided information on the number of days of missed work/education before IOS entry and during follow-up (43.7% (52/119) and 62.2% (74/119) of patients in Spain, and 39% (354/907) and 43.1% (391/907) of patients from other countries, respectively. Additionally, it is possible that the lesser severity of attacks experienced by patients in Spain may have influenced their perception of disease burden. Patients in Spain reported fewer hospitalizations before IOS entry, consistent with patients reporting fewer severe/very severe attacks.

Use of LTP was reported in two-thirds of patients from Spain compared with half of patients in other countries, possibly contributing to the finding of fewer hospitalizations in Spain. Trends in LTP use in IOS patients in Spain are consistent with previous findings from a single-center, observational, retrospective study conducted in 2010 [15]. In that study, androgens were used by nearly 70% of 45 patients receiving LTP; in the current analysis, of 73 patients who reported LTP use during follow-up in IOS, 39.7% used danazol and 43.8% used stanozolol (patients may be included in one or more categories). The single-center study also suggested that HAE-1/2 may be less severe in patients from Spain, indicated by use of lower than maximum recommended doses of androgens for LTP at time of treatment and follow-up. The present analysis provides additional evidence supporting this hypothesis; however, further investigation is needed to confirm these findings.

Although LTP use may have reduced frequency of attacks in both groups, LTP use did not reduce the proportion of severe/very severe attacks in patients from other countries or in Spain. There was a higher proportion of severe/very severe attacks in patients receiving LTP in other countries versus patients not receiving LTP (54.5% vs 39.7%). It is possible that patients who are known to have more frequent severe/very severe attacks are more likely to be prescribed LTP, and patients may be more likely to report severe/very severe attacks compared with mild attacks. In Spain, the proportion of severe/very severe attacks was similar in patients receiving LTP versus patients not receiving LTP (40.4% vs 42.3%). This difference may have resulted from greater use of androgens as LTP in Spain, or from the fact that severe/very severe attacks in Spain were already low. Although historical evidence suggests that androgen use at the lowest effective dose (to minimize risk of AEs) can achieve control and is acceptable in some patients, international guidelines generally recommend that androgens should be regarded critically, given their side effect profile and contraindications [1, 16]. Importantly, decisions related to LTP options should take into consideration patients’ individual needs, including the fact that androgen therapy is often the least expensive LTP option. Future studies that account for availability of newer LTP options with more favorable safety profiles are warranted to corroborate these findings.

Various country-specific IOS data have been published in recent years [17–19]. In Israel, patients were diagnosed earlier and had shorter time from onset to diagnosis and shorter total attack duration compared with other countries [19]. In the United Kingdom, higher rates of self-administration were observed, compared with other countries [17]. In Germany, patients reported significantly fewer severe/very severe attacks compared with other IOS countries as well as significantly shorter time to icatibant treatment, time to resolution, and total attack duration [18]. Patients from centers in Spain are the first country-specific group in IOS to report longer time to icatibant treatment, time to resolution, and total attack duration compared with patients from centers in other participating countries. Delayed use of icatibant may have resulted in longer time to resolution and total attack duration, consistent with observations that earlier treatment of HAE attacks is associated with improved outcomes [10]. Differences in icatibant use by country may be due to variations in health systems and health care resources as well as differing local practices and cultural variations.

Several limitations of this analysis should be considered, including the observational and retrospective nature of IOS and absence of prespecified analyses; potential for recall bias in patient data during follow-up visits; and propensity for incomplete/missing data and variable follow-up length in registries.

## Conclusions

Our findings may indicate differences in HAE management practices in Spain compared with other countries, or country-specific patient selection or reporting procedures. Education on the benefits of early on-demand treatment of HAE attacks is warranted to improve treatment outcomes.

## Supplementary Information

**Additional file 1: Table S1.** Employment status of patients from Spain compared with other countries at enrollment in IOS. **Fig. S1.** a Enrollment rate in IOS from 2009–2018 (cumulative patients) and b years of follow-up in IOS in Spain vs other countries. IOS, Icatibant Outcome Survey. **Fig. S2.** Median time to diagnosis, by year of birth, in patients from Spain vs other countries. **Fig. S3.** Proportions of patients with icatibant-treated attacks and treated attacks with other treatment per year in a Spain and b other countries. 2009 and 2019 were excluded because data were not collected for full years. Patients could be included in either treatment category. **Fig. S4.** Evolution per year of self-administered icatibant for the treatment of HAE attacks in patients in Spain vs other countries. 2009 and 2019 were excluded because data were not collected for full years. HAE, hereditary angioedema. **Fig. S5.** Icatibant treatment outcomes over time from IOS initiation in July 2009 to data cutoff in January 2019. Lines within box plots represent median time in hours, lower and upper lines of boxes represent the 25th and 75th percentiles, and box whiskers denote the minimum and maximum. **Fig. S6.** Time to icatibant treatment by patient/attack characteristics in patients in Spain vs other countries. **Fig. S7.** Proportions of patients reporting use of LTP medications per year in Spain vs other countries. 2009 and 2019 were excluded because data were not collected for full years. Counts and percentages are based on patient-reported LTP use during that year. The denominators count all patients in IOS follow-up at any time during the year who do not have LTP with start and end dates missing. IOS, Icatibant Outcome Survey; LTP, long-term prophylaxis.

## Data Availability

The datasets, including the redacted study protocol, redacted statistical analysis plan, and individual participants data supporting the results of the study, will be made available after the publication of study results within three months from initial request, to researchers who provide a methodologically sound proposal. The data will be provided after its de-identification, in compliance with applicable privacy laws, data protection, and requirements for consent and anonymization.

## Abbreviations

AE: Adverse event
HAE: Hereditary angioedema
HAE-1/2: Hereditary angioedema due to C1 inhibitor deficiency
IOS: Icatibant Outcome Survey
IQR: Interquartile range
LTP: Long-term prophylaxis
