# Supplementary material for: Hereditary angioedema due to C1 inhibitor deficiency: real-world experience from the Icatibant Outcome Survey in Spain
Source: Allergy Asthma Clin Immunol. 2021 Dec 29;17:137. doi: 10.1186/s13223-021-00641-3 (PMC8715569; doi:10.1186/s13223-021-00641-3)

# eSupplement

## eTable 1 Employment status of patients from Spain compared with other countries at enrollment in IOS

|  | **Spain**  **n = 119** | **Other Countries  n = 907** |
| --- | --- | --- |
| Employment status |  |  |
| Patients with data, n | 97 | 641 |
| Status, n (%) |  |  |
| Employed | 61 (62.9) | 357 (55.7) |
| Self-employed | 1 (1.0) | 36 (5.6) |
| Unemployed | 9 (9.3) | 46 (7.2) |
| Retired | 5 (5.2) | 70 (10.9) |
| Student | 21 (21.6) | 91 (14.2) |
| Homemaker | 5 (5.2) | 22 (3.4) |
| Leave of absence/sabbatical | 0 | 5 (0.8) |
| Preschool | 0 | 5 (0.8) |
| Other | 1 (1.0) | 25 (3.9) |

IOS, Icatibant Outcome Survey

**eFig. 1**  **a** Enrollment rate in IOS from 2009–2018 (cumulative patients) and **b** years of follow-up in IOS in Spain vs other countries. IOS, Icatibant Outcome Survey


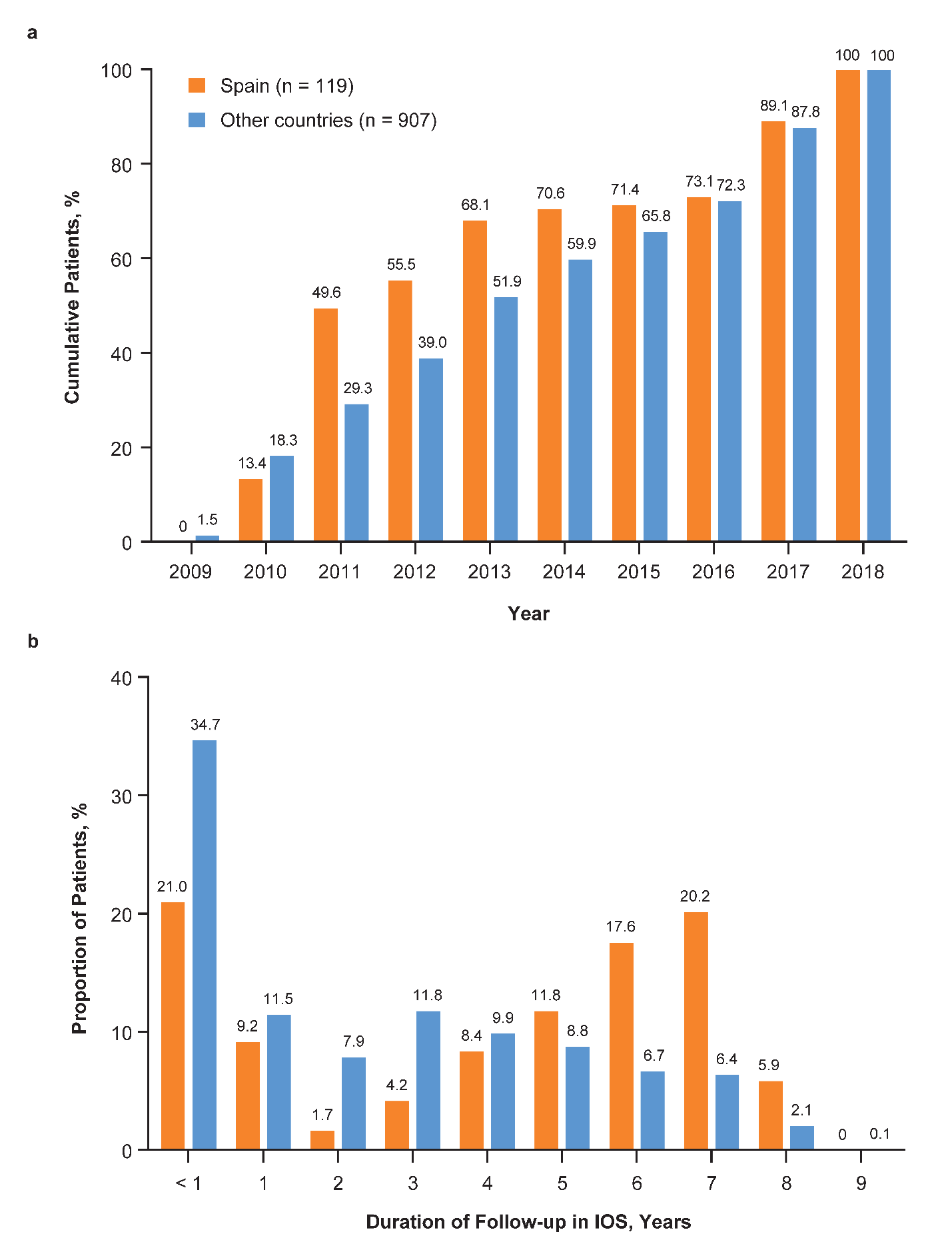


**eFig. 2**  Median time to diagnosis, by year of birth, in patients from Spain vs other countries
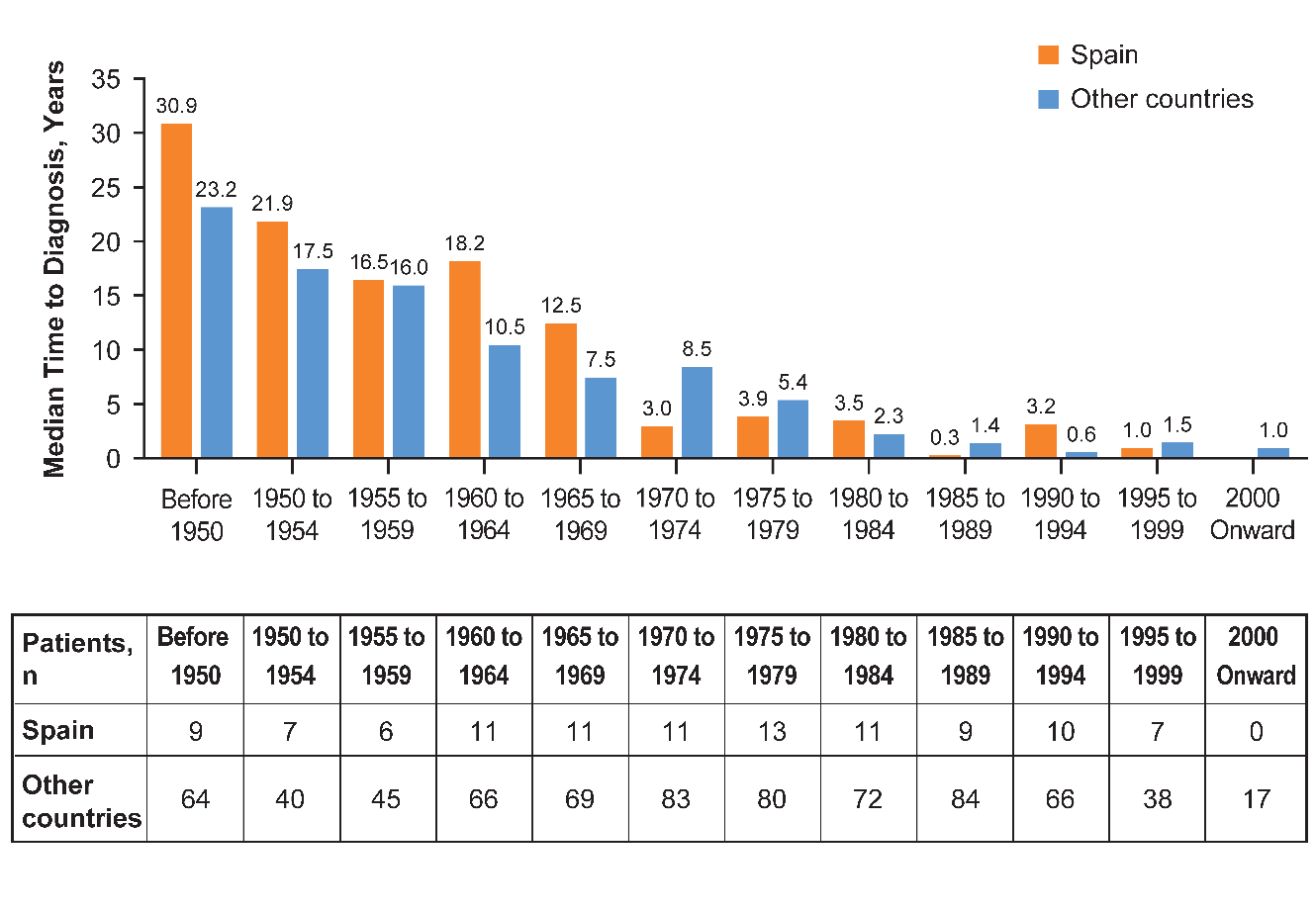


**eFig. 3**  Proportions of patients with icatibant-treated attacks and treated attacks with other treatment per year in **a** Spain and **b** other countries. 2009 and 2019 were excluded because data were not collected for full years. Patients could be included in either treatment category

##
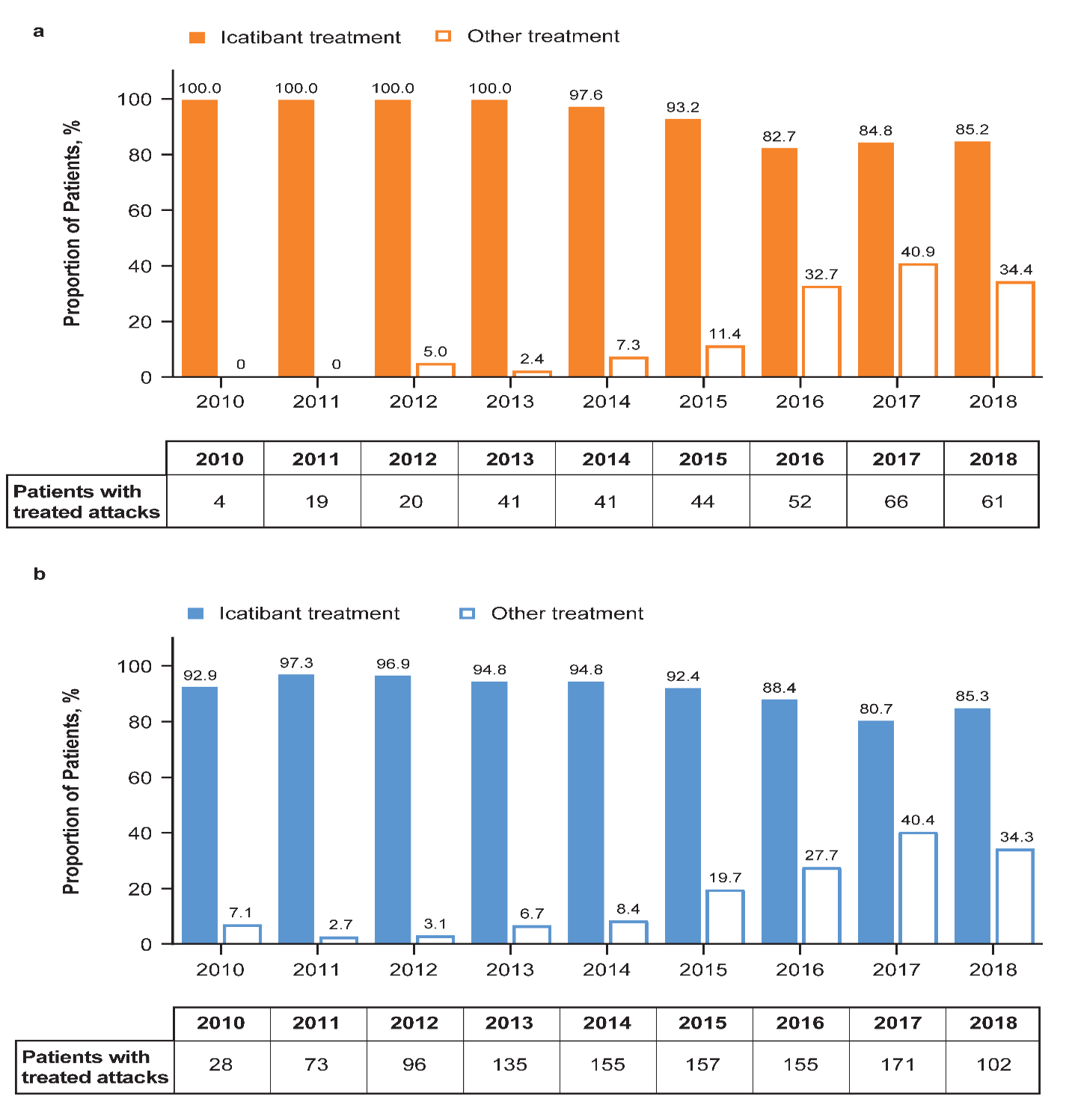


**eFig. 4**  Evolution per year of self-administered icatibant for the treatment of HAE attacks in patients in Spain vs other countries. 2009 and 2019 were excluded because data were not collected for full years. HAE, hereditary angioedema
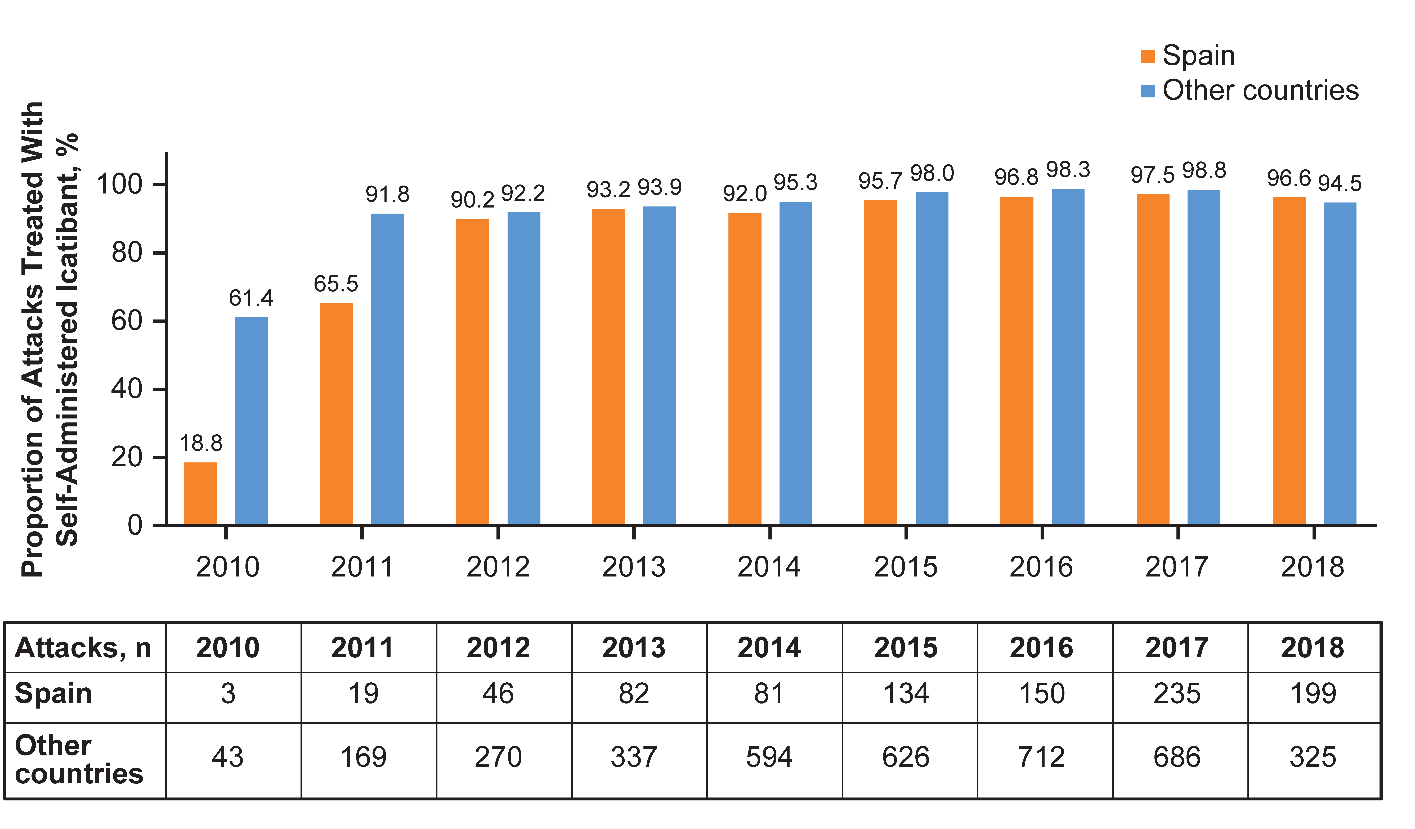


**eFig. 5**  Icatibant treatment outcomes over time from IOS initiation in July 2009 to data cutoff in January 2019. Lines within box plots represent median time in hours, lower and upper lines of boxes represent the 25th and 75th percentiles, and box whiskers denote the minimum and maximum


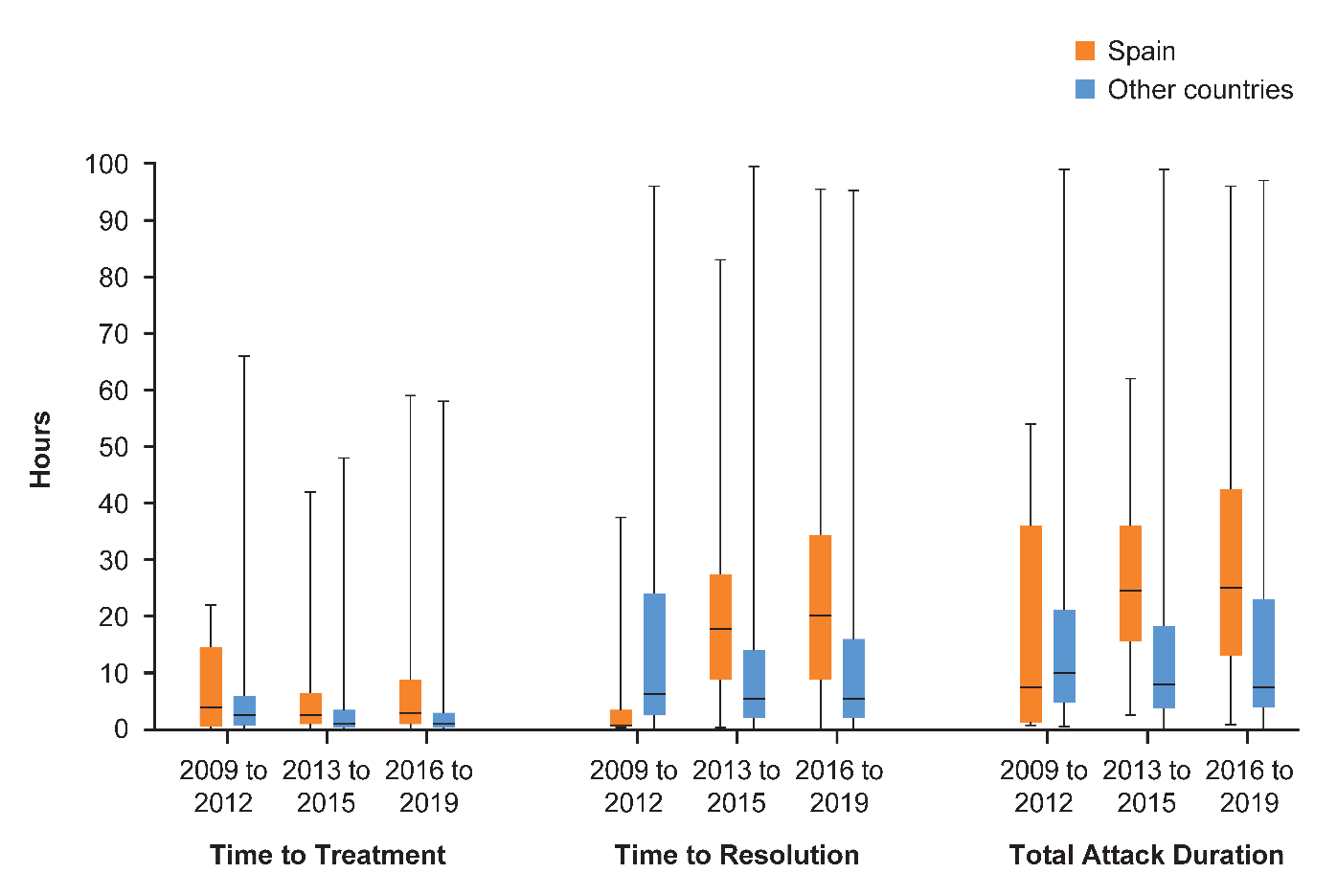


**eFig. 6**  Time to icatibant treatment by patient/attack characteristics in patients in Spain vs other countries


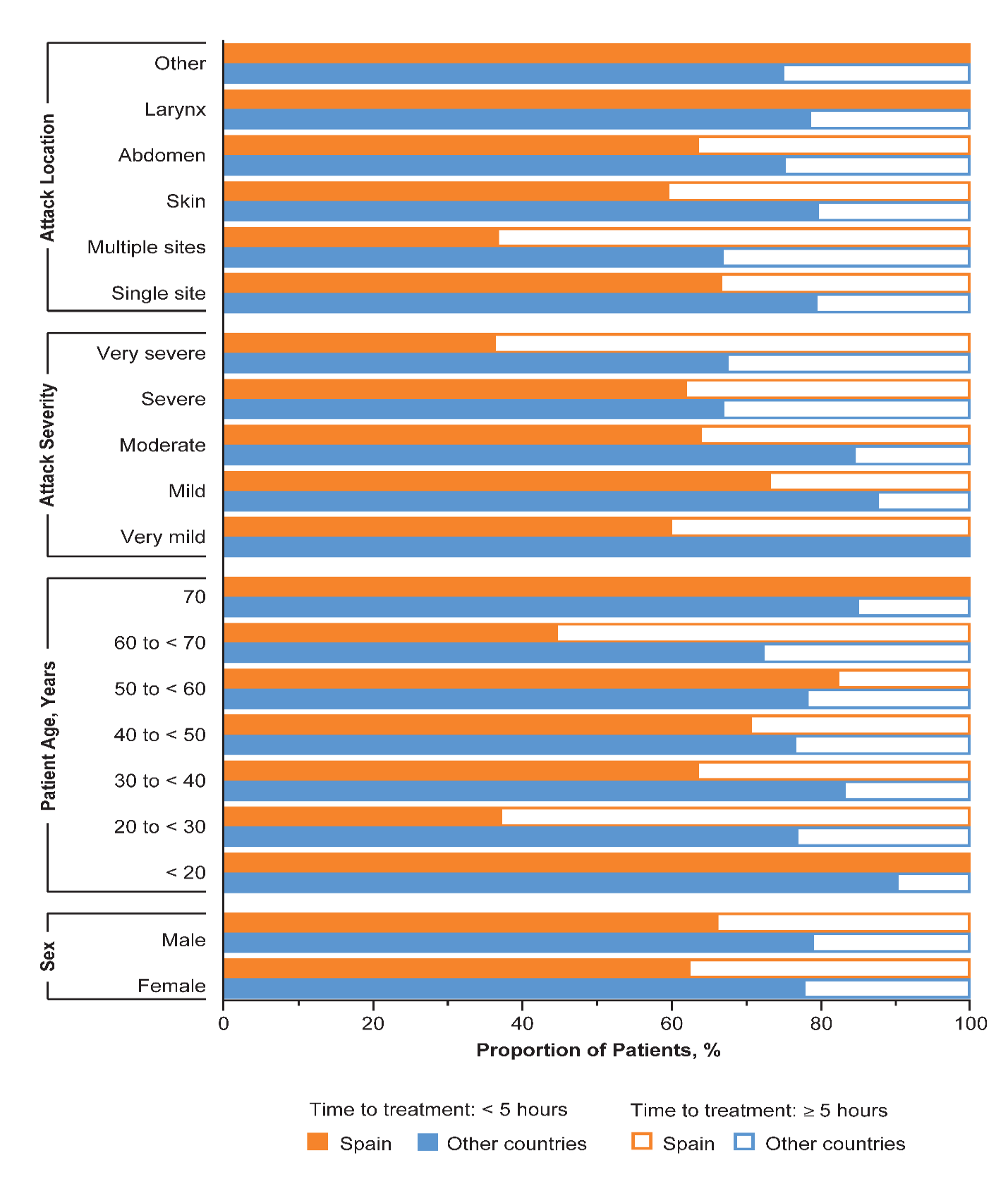


**eFig. 7**  Proportions of patients reporting use of LTP medications per year in Spain vs other countries. 2009 and 2019 were excluded because data were not collected for full years. Counts and percentages are based on patient-reported LTP use during that year. The denominators count all patients in IOS follow-up at any time during the year who do not have LTP with start and end dates missing. IOS, Icatibant Outcome Survey; LTP, long-term prophylaxis


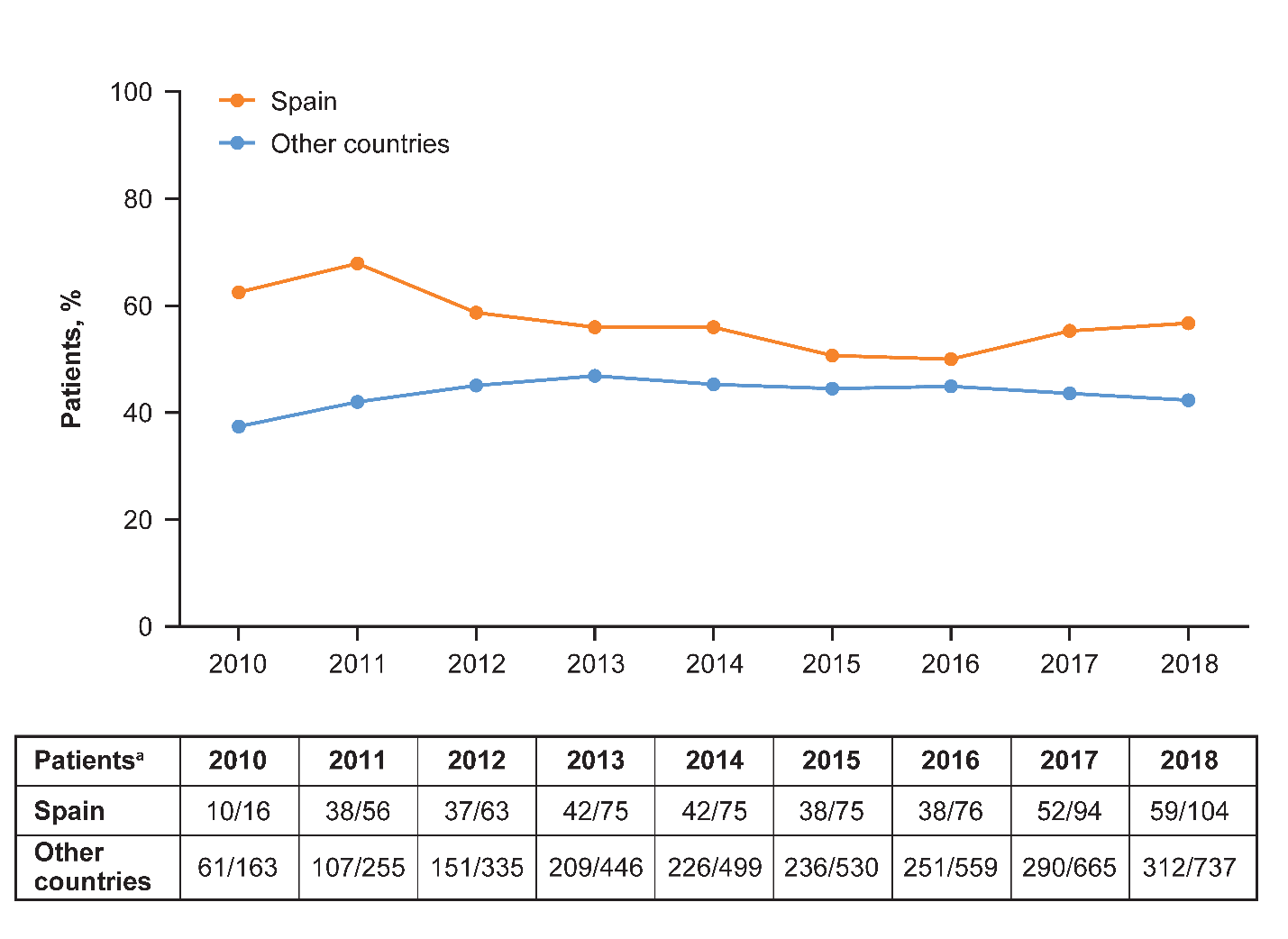

Supplement: Supplementary file 1 — Additional file 1: Table S1. Employment status of patients from Spain compared with other countries at enrollment in IOS. Fig. S1. a Enrollment rate in IOS from 2009–2018 (cumulative patients) and b years of follow-up in IOS in Spain vs other countries. IOS, Icatibant Outcome Survey. Fig. S2. Median time to diagnosis, by year of birth, in patients from Spain vs other countries. Fig. S3. Proportions of patients with icatibant-treated attacks and treated attacks with other treatment per year in a Spain and b other countries. 2009 and 2019 were excluded because data were not collected for full years. Patients could be included in either treatment category. Fig. S4. Evolution per year of self-administered icatibant for the treatment of HAE attacks in patients in Spain vs other countries. 2009 and 2019 were excluded because data were not collected for full years. HAE, hereditary angioedema. Fig. S5. Icatibant treatment outcomes over time from IOS initiation in July 2009 to data cutoff in January 2019. Lines within box plots represent median time in hours, lower and upper lines of boxes represent the 25th and 75th percentiles, and box whiskers denote the minimum and maximum. Fig. S6. Time to icatibant treatment by patient/attack characteristics in patients in Spain vs other countries. Fig. S7. Proportions of patients reporting use of LTP medications per year in Spain vs other countries. 2009 and 2019 were excluded because data were not collected for full years. Counts and percentages are based on patient-reported LTP use during that year. The denominators count all patients in IOS follow-up at any time during the year who do not have LTP with start and end dates missing. IOS, Icatibant Outcome Survey; LTP, long-term prophylaxis. [file 13223_2021_641_MOESM1_ESM.docx]
